# Synthesis, structure, and mechanical properties of silica nanocomposite polyrotaxane gels

**DOI:** 10.3762/bjoc.11.238

**Published:** 2015-11-16

**Authors:** Kazuaki Kato, Daisuke Matsui, Koichi Mayumi, Kohzo Ito

**Affiliations:** 1 Department of Advanced Materials Science, Graduate School of Frontier Sciences, The University of Tokyo, 5-1-5 Kashiwanoha, Kashiwa, Chiba 277-8561, Japan

**Keywords:** cyclodextrin, gel, mechanical property, nanocomposite, polyrotaxane

## Abstract

A significantly soft and tough nanocomposite gel was realized by a novel network formed using cyclodextrin-based polyrotaxanes. Covalent bond formation between the cyclic components of polyrotaxanes and the surface of silica nanoparticles (15 nm diameter) resulted in an infinite network structure without direct bonds between the main chain polymer and the silica. Small-angle X-ray scattering revealed that the homogeneous distribution of silica nanoparticles in solution was maintained in the gel state. Such homogeneous nanocomposite gels were obtained with at least 30 wt % silica content, and the Young’s modulus increased with silica content. Gelation did not occur without silica. This suggests that the silica nanoparticles behave as cross-linkers. Viscoelastic measurements of the nanocomposite gels showed no stress relaxation regardless of the silica content for <20% compression strain, indicating an infinite stable network without physical cross-links that have finite lifetime. On the other hand, the infinite network exhibited an abnormally low Young’s modulus, ~1 kPa, which is not explainable by traditional rubber theory. In addition, the composite gels were tough enough to completely maintain the network structure under 80% compression strain. These toughness and softness properties are attributable to both the characteristic sliding of polymer chains through the immobilized cyclodextrins on the silica nanoparticle and the entropic contribution of the cyclic components to the elasticity of the gels.

## Introduction

Nanocomposite materials, in which nanoparticles are distributed via a matrix such as resin or rubber, exhibit various functions that the matrix material cannot achieve by itself. For instance, polyurethane/magnetic nanoparticle nanocomposite elastomers permitted a wide range of elasticity modulation by controlling the magnetic field [1], owing to the alignment of nanoparticles within the polymer network. In addition to such functionalization, nanocomposition can effectively reinforce materials; a typical example is natural rubbers reinforced by nanoparticles such as carbon black and silica for practical use as tire materials. Because of adhesion of polymer chains to the nanoparticle surfaces, the chain mobility at the interface is considerably suppressed, thereby increasing the elastic modulus of the nanocomposite [2–6]. Although the nanocomposite strategy is promising for the design of relatively hard functional materials, it is not easy to realize soft nanocomposite materials because of the strong interactions at the interface that reduce the polymer chain mobility. For dielectric elastomers expected to be used as actuators and electric generating systems, nanocomposites should satisfy both a high dielectric constant that is assured by the nanoparticles and a low elastic modulus [7].

Mechanically interlocked supramolecular polymers, such as polyrotaxane [8–9], can control the interface between the matrix polymer and the nanoparticles. Polyrotaxane comprising an end-capped backbone polymer and threaded cyclic molecules such as cyclodextrins (CDs) can form a network structure by intermolecular binding of the cyclic components [10]. Since the polymer chains are topologically connected to each other without chemical bonds, the chains can slide through the cross-links and lead to several unique properties [11–16]. Polyrotaxanes were also applied for surface modification of substrates by attaching the cyclic components to the surface [17]. These results were based on the indirect connection among different polymers or between polymers and surfaces, so that the mobility of the polymers can be maintained at the cross-linking points or the interface.

**Figure 1:**
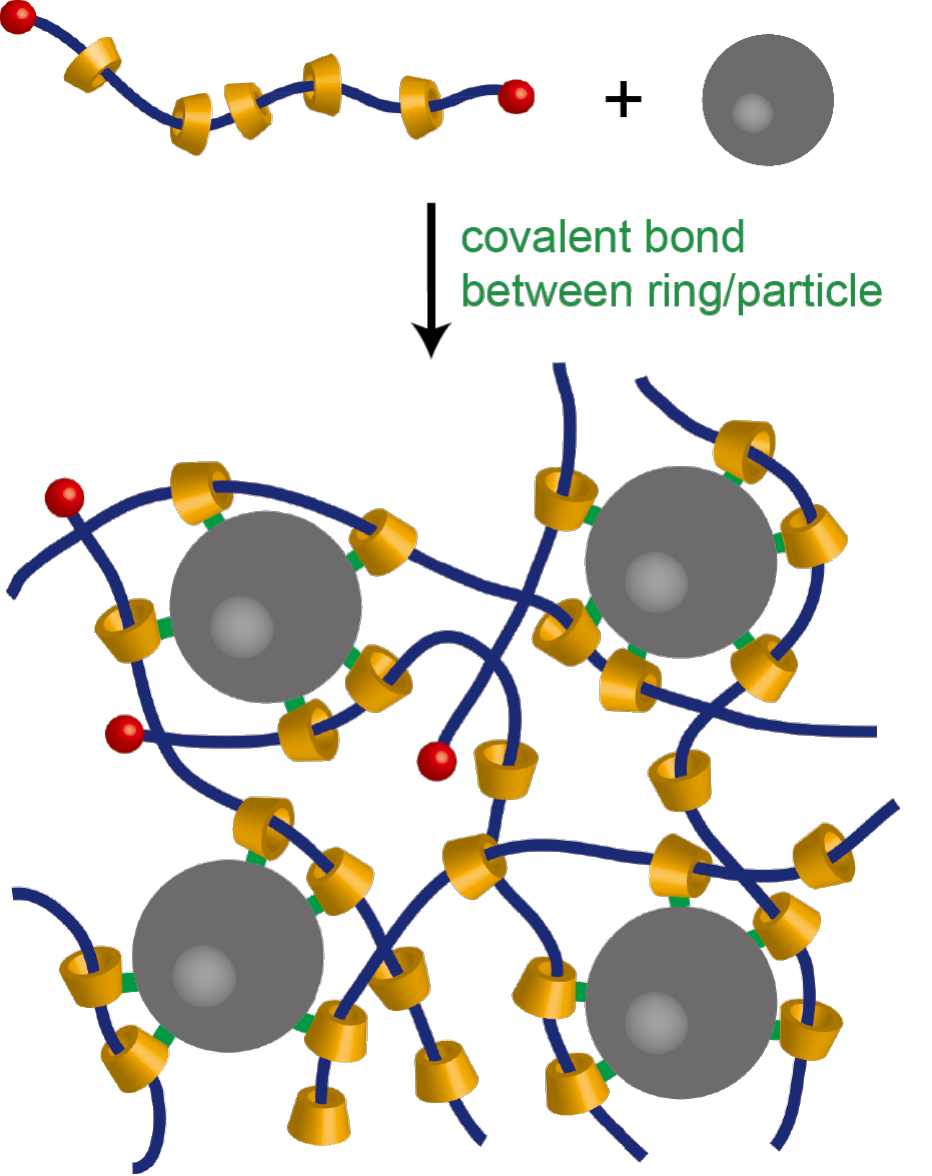
Nanocomposite polyrotaxane network formed by covalent bonds between cyclic components of polyrotaxane and the nanoparticle surfaces.

We demonstrate here that the indirect connection between the backbone polymers and nanoparticles can be applied also for soft nanocomposite materials. As illustrated in Figure 1, formation of chemical bonds between the cyclic components of polyrotaxanes to the surface of a nanoparticle may result in a network structure where the nanoparticle acts as a cross-linker. As the first model system, we employed a silica nanoparticle and a CD-based polyrotaxane whose rings were chemically modified to react with the surface of silica. The inner structure and the mechanical properties of the silica nanocomposite polyrotaxane gels were studied by small-angle X-ray scattering and viscoelastic measurements, respectively.

## Results and Discussion

**Scheme 1:**
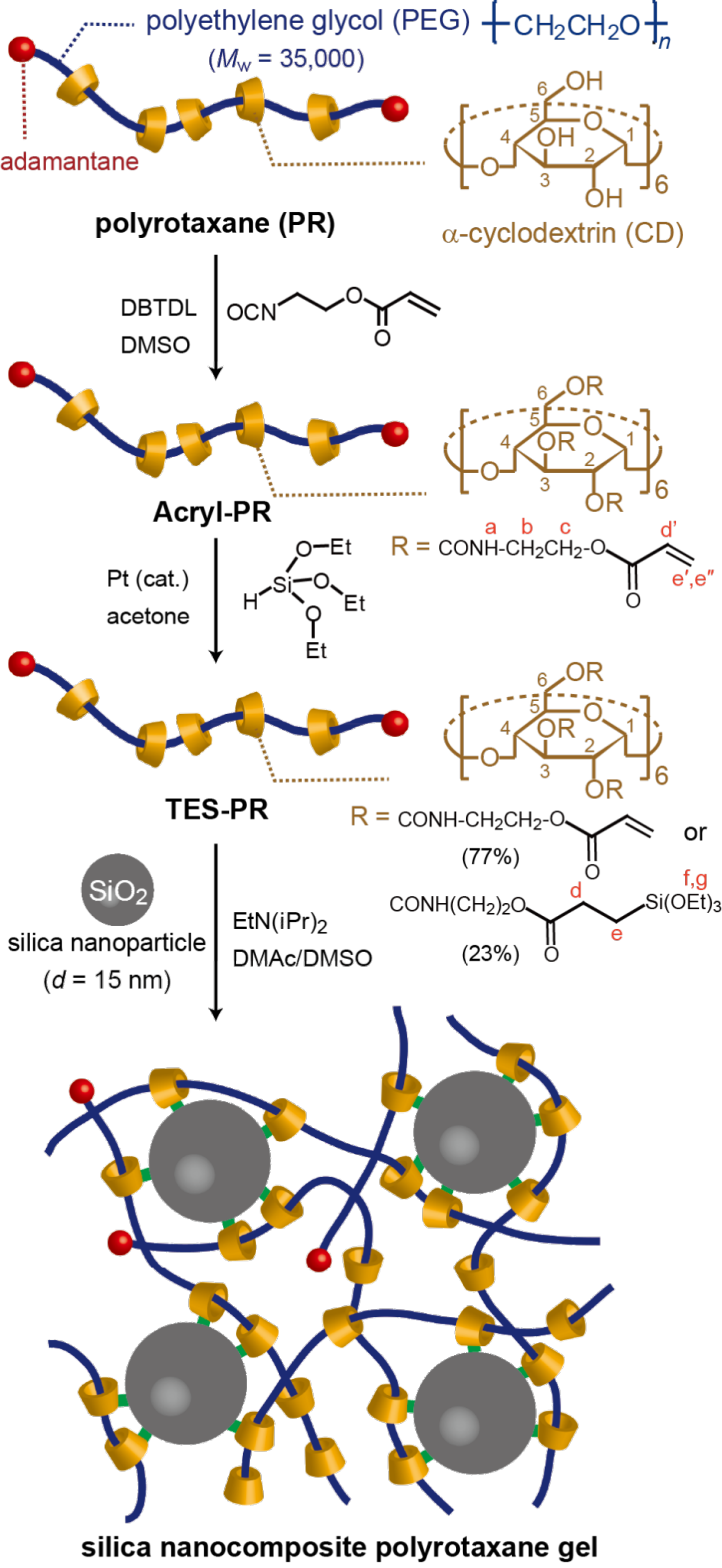
Synthesis of polyrotaxane with partially triethoxysilylated α-CDs (TES-PR) and crosslinking of the polyrotaxane solution via the reaction between the CDs and the surface of silica nanoparticles.

**Figure 2:**
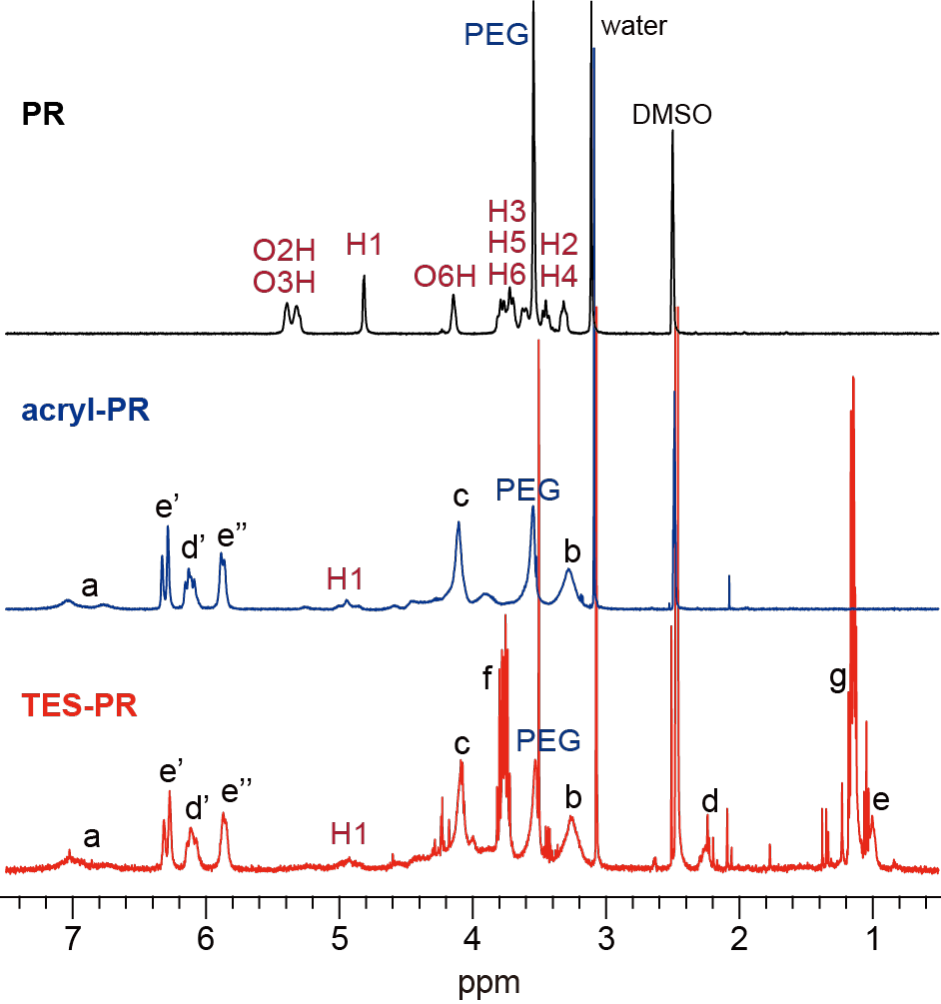
^1^H NMR spectra (400 MHz, DMSO-*d*_6_, 343 K) of polyrotaxane and its derivatives with modified cyclic components.

**Figure 3:**
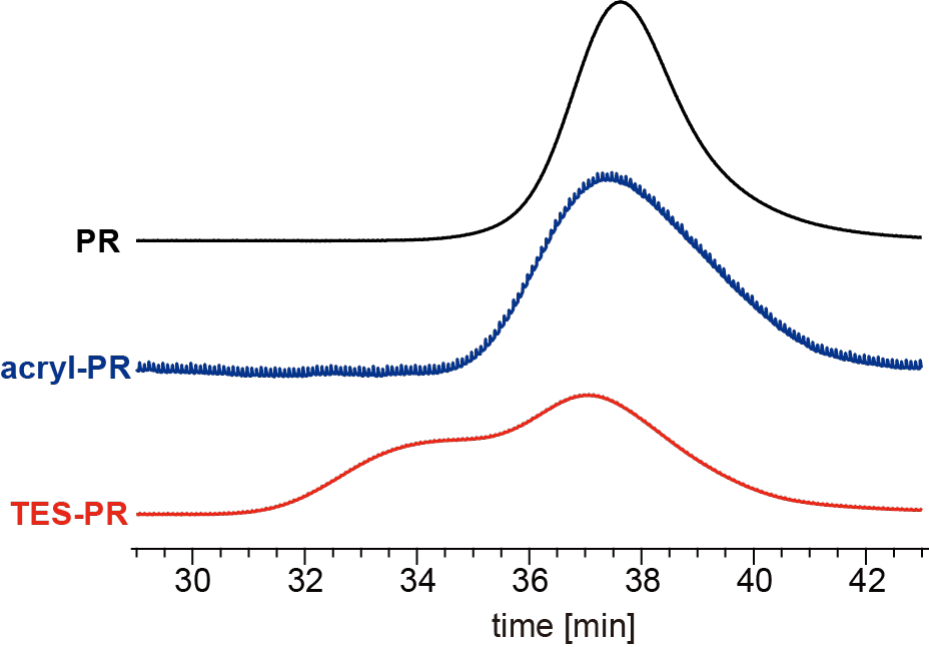
GPR traces of polyrotaxane and its derivatives; eluent: DMSO/LiBr (10 mM LiBr), detection: RI.

To achieve a covalent bond between the cyclic components (CDs, in this study) and the nanoparticle surfaces (SiO_2_), hydroxy groups of the cyclic components of a polyrotaxane comprised of α-CD and PEG (PR) was modified as shown in Scheme 1. The polyrotaxane with triethoxysilylated CD (TES-PR) is suitable for the reaction with SiO_2_ in the presence of base. Although TES-PR can be isolated just by vacuum drying to evaporate the reactant and solvent, the solid isolated in this way becomes insoluble in any solvent. This is due to the crosslinking induced by reactions between CDs. Thus, DMSO was added to the reaction solution first, followed by drying the solution under vacuum to eliminate acetone and reactant; as a result, a pure DMSO solution of TES-PR was obtained. The solution was used for the following characterization. Figure 2 shows the ^1^H NMR spectra of TES-PR, PR, and an intermediate polyrotaxane with acryloyl groups at the CDs (Acryl-PR). All peaks were assigned as shown. From these integral values, the modification degree of Acryl-PR was calculated to almost 100% (corresponding to 18 acryloyl groups in each CD), with only 23% of the acryloyl groups reacted with triethoxysilane to generate TES-PR. GPC traces shown in Figure 3 indicate that the interlocked structure was retained throughout the modification and that the molecular weight of PR was increased with each modification step. The GPC trace of TES-PR also indicated the existence of high-molecular weight TES-PR. This probably multimeric TES-PR is the result of intermolecular reactions between triethoxysilyl groups during hydrosilylation. Since the pure TES-PR DMSO solution was stable at room temperature, the solution was used for the following reaction with silica nanoparticles.

**Figure 4:**
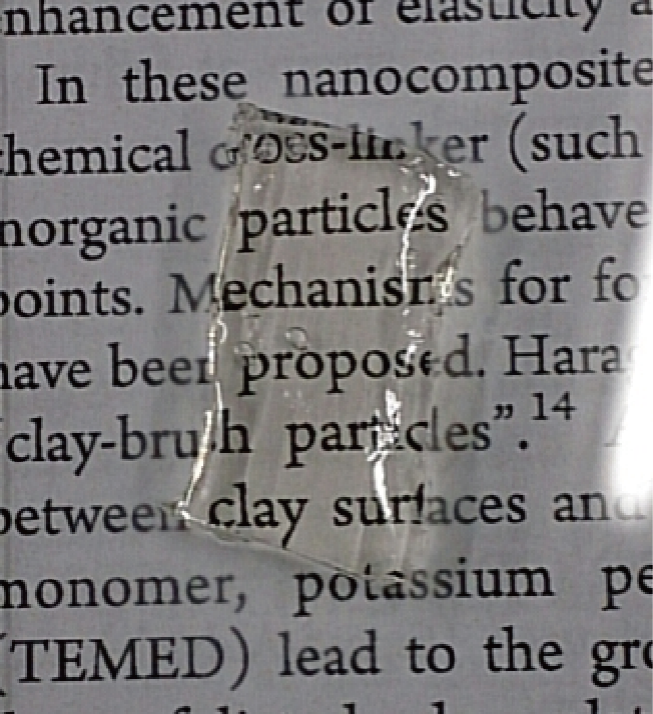
Photograph of silica nanocomposite polyrotaxane gel.

A TES-PR solution in DMSO was mixed with a dispersed solution of silica nanoparticles with 15 nm diameter in *N*,*N*-dimethylacetamide (DMAc) (20 wt %), followed by the addition of diisopropylethylamine to initiate the reaction between TES-PR and silica. The pre-gel solution was transferred to a glass mold with 3 mm thickness and cured for 16 h at 373 K. As shown in Figure 4, the obtained gel was transparent, indicating no significant aggregation. Five gels were synthesized in the same way with different initial concentrations of silica nanoparticles. Notably, in the absence of silica nanoparticles, gelation did not occur; only a slight increase of viscosity was observed. This result clearly shows that the gelation was achieved mainly by the reaction between silica and TES-PR, with a parallel minor reaction occurring between the triethoxysilyl groups of different TES-PRs.

**Figure 5:**
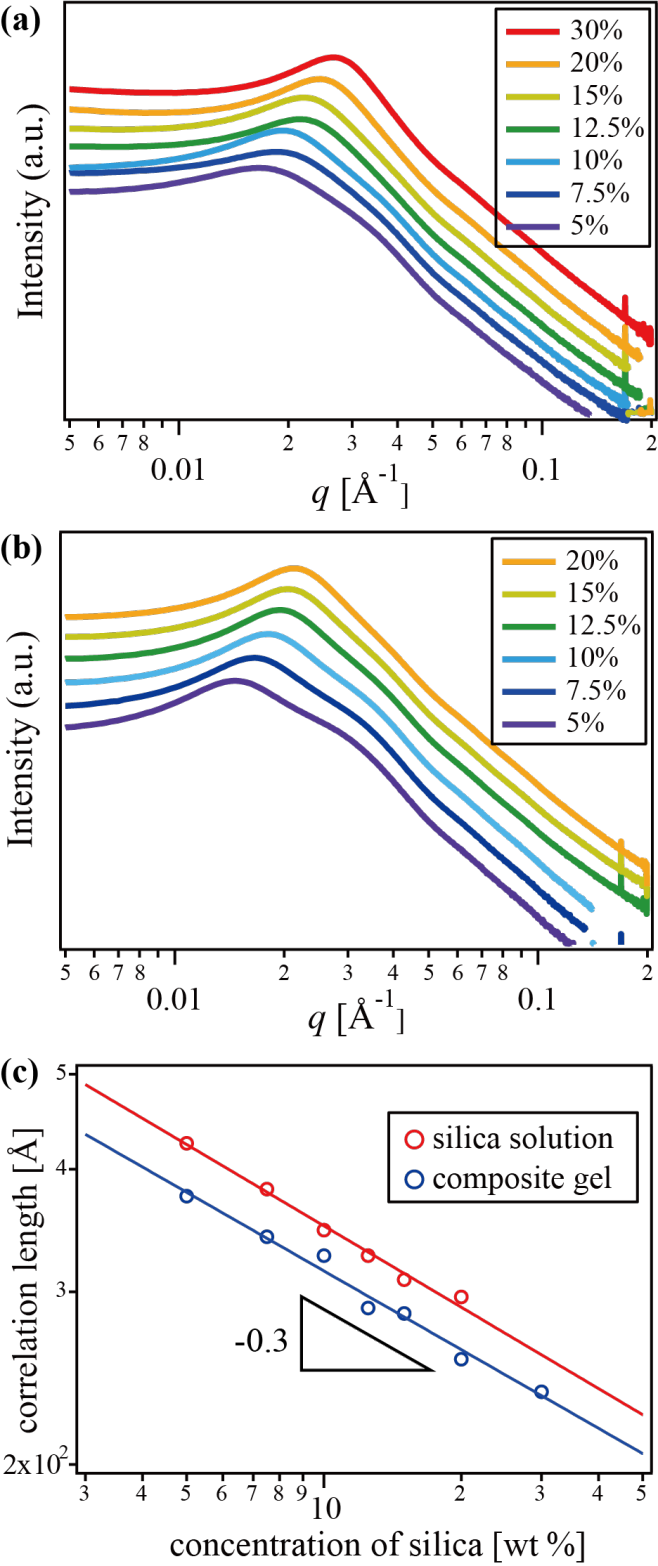
SAXS profiles of (a) silica nanocomposite polyrotaxane gels and (b) silica solutions with different concentration of silica; (c) double logarithmic plots of the correlation length against silica concentrations.

To elucidate the dispersity of silica nanoparticles in the gels, small-angle X-ray scattering (SAXS) was carried out. Figure 5a shows the SAXS profiles of silica nanocomposite gels with different silica concentrations. A Bragg’s peak around *q* = 0.01–0.05 Å^−1^ was observed, with the peak shifting toward higher *q* with increasing silica concentration. It is noteworthy that no increase of scattering intensity toward the low *q* limit, indicating that no aggregation is seen even in that *q* range. Similar SAXS profiles were observed in silica solutions (Figure 5b). Since the correlation distance becomes shorter with the increase of concentration, the distance is thought to be the separation between silica nanoparticles. When we assume homogeneous distribution of particles, the following relation exists in the distance between particles *d* and the concentration *c*:

[1]
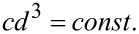


Figure 5c shows the double log arithmic plots of correlation length, which is obtained from the *q* of the peak top (*d* = 2π/*q*), against silica concentration for the nanocomposite gels and silica solutions. In both cases, a similar power dependence, 
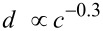
, as in Equation 1, was obtained. Therefore, similarly to the silica solution, the homogeneous dispersion of silica nanoparticles was retained in the nanocomposite gels until at least 30 wt % silica content.

**Figure 6:**
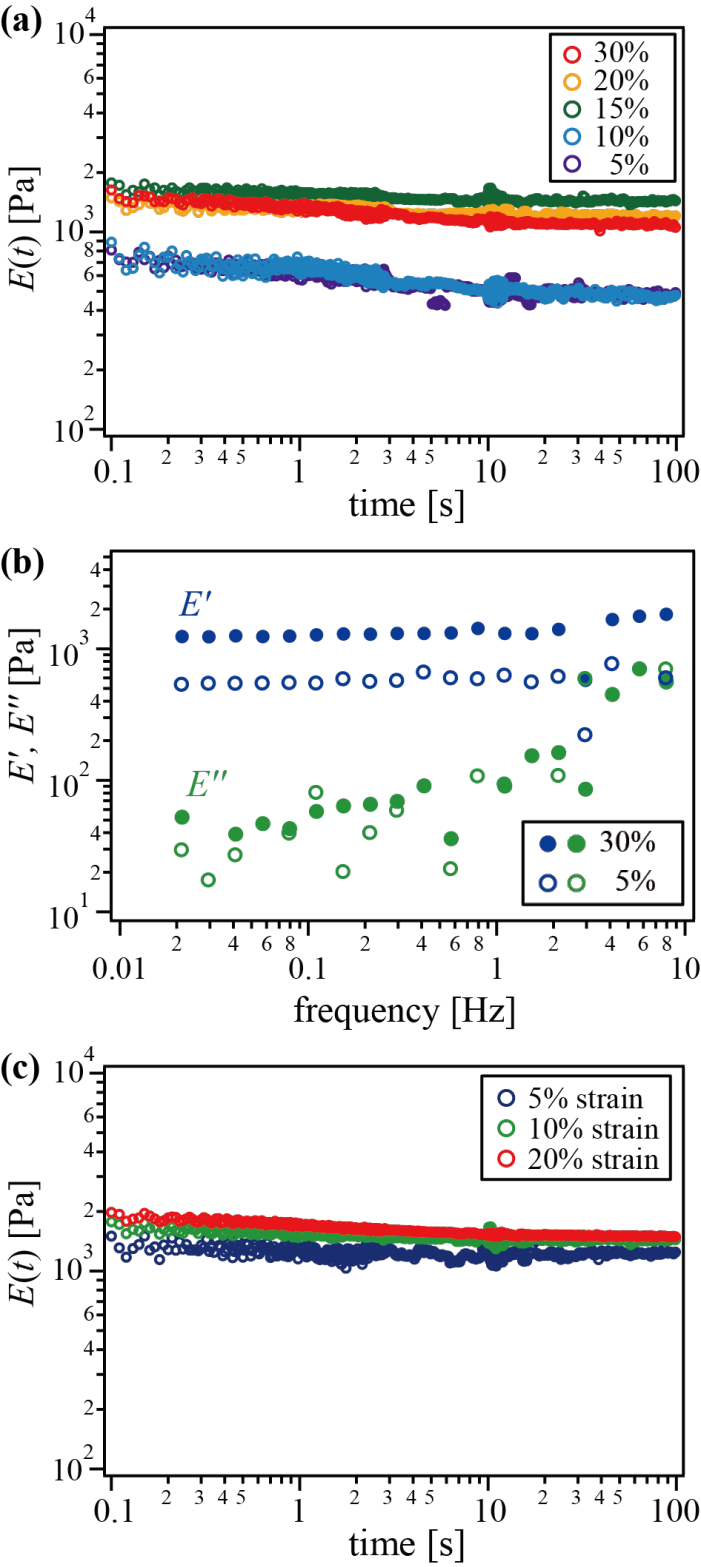
(a) Relaxation Young’s modulus with 5% strain and (b) frequency-dependence of dynamic storage and loss moduli for silica nanocomposite polyrotaxane gels with different silica concentrations; (c) strain-dependence of relaxation Young’s modulus for the gel with 15% silica.

Viscoelastic measurements were carried out for the obtained gels. Figure 6a shows the results of stress relaxation tests. At high silica concentration (more than 15%), the Young’s modulus is about 1 kPa, whereas a lower silica concentration yielded a lower modulus of about 0.5 kPa. In addition, as mentioned above, no gelation occurred without silica. Thus, the modulus tends to increase with the concentration of silica, though the incremental increase of modulus is not proportional to silica concentration but stepwise. This suggests that the cross-linking density becomes higher with increasing silica concentration because the silica acts as the polyrotaxanes cross-linker. The results of dynamic viscoelastic measurements shown in Figure 6b also suggest the network formation via silica nanoparticles. Dynamic storage Young’s modulus *E*′ was increased with silica content and the modulus was consistent with *E*(*t*). On the other hand, the loss modulus *E*″ hardly changed with increasing of silica concentration. Thus, the ratio of the loss modulus to storage modulus, the so-called loss tangent, decreased with silica concentration. This typical tendency generally observed in chemical gels that is attributed to the decrease of dangling chains, resulting in the formation of a denser network. In addition, there was no stress relaxation with finite equilibrium modulus regardless of the silica content. Figure 6c shows the strain-dependence of the relaxation Young’s modulus for the gel with 15% silica. The modulus was almost independent of the strain. These results clearly indicate that the silica nanocomposite polyrotaxane gels form an infinite network structure with negligible physical cross-links that have finite lifetime to exhibit stress relaxation, similar to ideal chemical gels. At the same time, the negligible relaxation indicates that almost all silica nanoparticles were bound to the polymer network.

However, the Young’s modulus of the nanocomposite gels is abnormally low. From the Young’s modulus, *E*, with an assumption of ideal polymer network, the averaged molecular weight between cross-links, *M*_x_, can be estimated by the following equation:

[2]
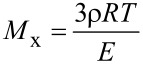


where ρ is the density of polymer, *R* the gas constant, and *T* the absolute temperature. *M*_x_ of the representative nanocomposite polyrotaxane gels with *E* = 1 kPa was calculated to be 7 × 10^5^ g/mol. This *M*_x_ is considerably larger than the molecular weight of the precursor polyrotaxane, TES-PR (~2 × 10^5^ g/mol). Thus, this result gives us an unlikely picture for the network where several polyrotaxanes are bound to form a single partial chain. This discrepancy probably arises from the assumption of the validity of rubber elastic theory for these gels. Surely, for most of composite gels or rubbers, the theory is already invalid due to the presence of strong interactions between polymers and embedded particles in addition to the covalent crosslinking points. However, such interactions increase not, decrease, modulus. Thus, the interactions between the polyrotaxane and silica cannot explain the significantly low modulus.

Recent research in polyrotaxane gels, which were obtained by the simple cross-linking of polyrotaxanes via intermolecular covalent bonds between CDs, indicated a new origin of the elasticity experimentally and theoretically [12,14,16,18]. Since the polymer chains can slide through the cross-links, the anisotropic orientation of chain segments, which causes the entropic elasticity of rubbers and gels, can be relaxed. Simultaneously, the relaxation of chains results in inhomogeneous distribution of the threaded CDs. As the CDs continuously slide along the backbone polymer, their inhomogeneous distribution can generate entropic stress. Thus, the origin of elasticity is not the entropy of polymer chains but that of the CDs, and the abnormally small modulus of polyrotaxane gels can be explained by the characteristic elasticity. Similarly, the extremely low modulus of the nanocomposite polyrotaxane gels is attributable to the characteristic elasticity originated from the entropy of CDs. Since the infinite network was formed mainly by the covalent bonds between CDs and silica surface, the polymer chains are bound to neither CDs nor silica. If the polymer chains are directly attached to the silica surface, the chain mobility were drastically decreased, yielding an increased modulus as observed in conventional composite gels and rubbers. Therefore, the polymer chains slide through the immobilized CDs on the surface of silica, and thus the unique properties observed in polyrotaxane gels may also appear in these nanocomposite polyrotaxane gels.

**Figure 7:**
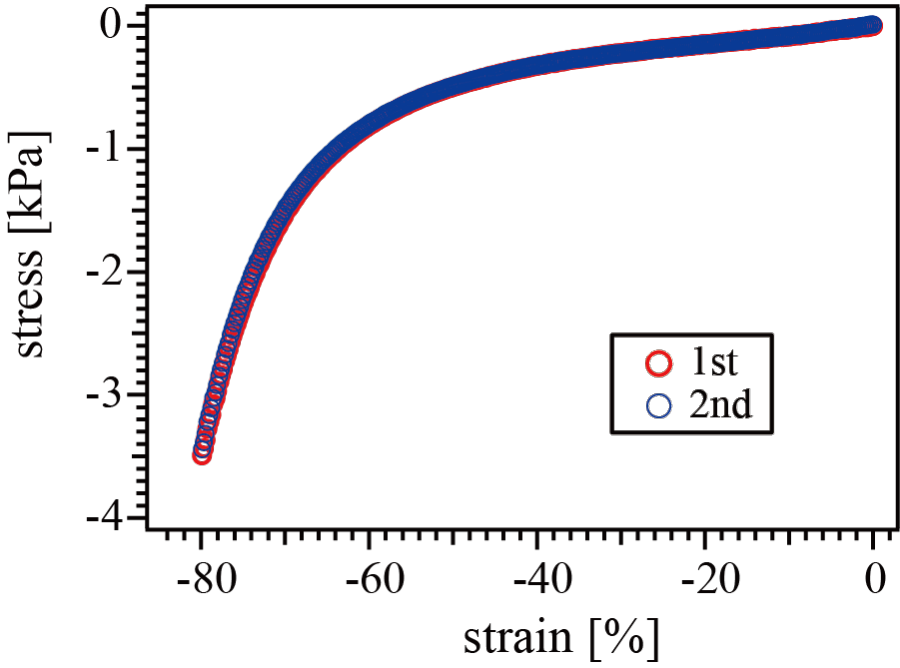
Repeated stress-strain curves for a silica nanocomposite polyrotaxane gel with 20% silica content.

In addition to the characteristic softness, the nanocomposite gels exhibited significant toughness. Figure 7 shows the stress–strain curves of one of the nanocomposite gels under compression. Since the fracture point is not always clear in the stress–compression behavior, the same measurement was repeated using the same gel. The two curves are in complete agreement, indicating that no fracture occurred during the first compression. This data proved that the gel is compressive to at least one-fifth of the original thickness without fracture or network structure recombination. The toughening mechanism may be essentially the same as that in polyrotaxane gels: the stress applied to chains can be distributed by chain sliding through the immobilized CDs on the silica surface, the so-called pulley effect [11]. In this way, the silica nanocomposite polyrotaxane gels exhibited two unique properties: soft and tough, through the novel method for nanocomposites without chemical bonds or significant interactions between polymers and nanoparticles.

## Conclusion

Here we demonstrate the synthesis of a novel nanocomposite gel where the polymer chains were not directly bonded but rather mechanically interlocked to the silica surfaces. The silica nanoparticles were homogeneously distributed in the gel and worked as cross-linkers to immobilize the cyclic components of the polyrotaxanes on the silica surfaces. As the backbone polymer chains were not only free from adhesion to the silica surface but can also slide through the immobilized cyclic components, the nanocomposite gel achieved low Young’s modulus and high toughness without any detectable fracture or recombination of network structure under 80% compression. These results suggest that the concept of topological cross-linking previously studied with polyrotaxane gels is applicable to other nanocomposite materials, though our model system utilized silica nanocomposites. Functionalization and applications of the nanocomposite polyrotaxane gels with other nanoparticles are now in progress.

## Experimental

### Materials

Crude polyrotaxane consisting of polyethylene glycol (PEG, *M*_n_ = 32,000) and α-cyclodextrin (CD) were purchased from Advanced Softmaterials, Inc. The crude polyrotaxane was purified by repeated reprecipitation from its DMSO solution into deionized water. The obtained precipitate was freeze-dried and the refined polyrotaxane (PR) obtained as a white powder. The coverage, which is a measure how densely the backbone PEG is covered with CDs, was calculated to be 25% based on the ^1^H NMR spectrum. Standard polymers for the calibration of the molecular weights by size-exclusion chromatography (SEC) were purchased from Polymer Source, Inc. Solutions of silica nanoparticle with 15 nm of diameter in *N*,*N*-dimethylacetamide was kindly supplied by Nissan Chemical Industries, Ltd. 2-Isocyanatoethyl acrylate was purchased from Showa Denko K.K. Xylene solutions of platinum(0)-1,3-divinyl-1,1,3,3-tetramethyldisiloxane (~2%) was purchased from Sigma-Aldrich Corporation. All other chemicals were purchased from Tokyo Chemical Industry Co., Ltd., or Wako Pure Chemical Industries, Ltd., and all reagents were used as received without further purification except for PR.

### Characterization measurements

^1^H NMR spectra (400 MHz) were recorded on a JEOL JNM-AL400 spectrometer at 343 K. The chemical shift was calibrated using DMSO (2.50 ppm) as an internal standard. SEC was performed on TOSOH HLC-8220 with two Shodex OH Pack SB-806MHQ columns, with DMSO at 50 °C in the presence of 0.01 M lithium bromide as the eluent using RI detection and PEG standards. The flow rate was 0.4 mL/min.

### Synthesis of acryloyl modified polyrotaxane (Acryl-PR)

Three grams of PR previously dried under vacuum was dissolved in anhydrous DMSO (60 mL). 2-Isocyanatoethyl acrylate (9 mL) and dibutyltin dilaurate (90 μL) were added and stirred at room temperature for 5 days. The product was precipitated by pouring the reaction solution into methanol. The precipitate was repeatedly washed with methanol and then dried. The dried product was again dissolved in acetone and then a large amount of methanol was added to precipitate the product. This process was repeated again followed by drying to yield acryloyl modified polyrotaxane (Acryl-PR, 5.41 g) as a white solid: ^1^H NMR (400 MHz, DMSO-*d*_6_, 343 K): 7.0 (NH of urethane), 6.3, 5.9 (CH_2_ of vinyl), 6.1 (CH of vinyl), 4.9 (C_(1)_H of CD), 4.1 (C(=O)O-CH_2_), 3.5 (PEG), 3.2 (-CH_2_-NH).

### Synthesis of triethoxysilyl modified polyrotaxane (TES-PR)

Two grams of Acryl-PR previously dried under vacuum was dissolved in anhydrous acetone (40 mL). Triethoxysilane (600 μL) and a xylene solution of platinum(0)-1,3-divinyl-1,1,3,3-tetramethyldisiloxane dibutyltin dilaurate (200 μL) were added followed by refluxing at 65 °C overnight. The reaction solution was then centrifuged to precipitate a minor insoluble component, and the supernatant collected. Because the product became insoluble in any solvent once it was completely dried, the crude acetone solution was stored at room temperature until just before use. For further characterization, DMSO or DMSO-*d*_6_ was added to the acetone solution, and then the solution was dried under vacuum to evaporate unreacted reactants and non-DMSO solvent. As a result, pure solutions in DMSO or DMSO-*d*_6_ were obtained because DMSO has a sufficiently high boiling point to resist evaporation. ^1^H NMR (400 MHz, DMSO-*d*_6_, 343 K): 7.0 (NH of urethane), 6.3, 5.9 (CH_2_ of vinyl), 6.1 (CH of vinyl), 4.9 (C_(1)_H of CD), 4.1 (C(=O)O-CH_2_), 3.8 (SiO-CH_2_), 3.5 (PEG), 3.2 (-CH_2_-NH), 2.3 (OC(=O)-CH_2_), 1.1 (SiOCH_2_-CH_3_), 1.0 (Si-CH_2_).

### Synthesis of silica nanocomposite polyrotaxane gels

1.5 mL of DMSO was added to 3 mL of the crude acetone solution of TES-PR, whose silica content was known to be 5 wt %/vol. The solution was dried under vacuum to eliminate impurities, yielding 1.5 mL of 0.1 g/mL TES-PR solution in DMSO. A DMAc solution of silica nanoparticles was added to the TES-PR solution, and then the mixed solution was dried under vacuum to evaporate DMAc, resulting in 1.5 mL of TES-PR and silica pre-gel solution in DMSO. By changing the added volume of silica solution, five pre-gel solutions with different silica contents were obtained. Diisopropylethylamine (15 μL) was added to the pre-gel solution, and then the solution was transferred into a Teflon/glass mold with a 25 mm × 25 mm × 3 mm void. The reaction was carried out at 100 °C for 16 h to yield a transparent nanocomposite gel. In the same way, thinner gels were also prepared in a mold with a 20 mm × 20 mm × 1 mm void for the following structural analysis.

### Small-angle X-ray scattering measurement

Synchrotron small-angle X-ray scattering (SAXS) experiments were carried out at the beamline 6A of Photon Factory, HighEnergy Accelerator Research Organization, KEK (Tsukuba, Japan). The wavelength λ of the incident X-ray beam was 1.50 Å and the beam size was 0.5 mm (vertical) × 0.5 mm (horizontal). PILATUS 300k (Dectris) was used to record SAXS patterns. The sample-to-detector distance was 2.6 m and the sample thickness was 1 mm. The exposure time for each sample was kept constant at 5 s. The scattering angle θ was calibrated by the diffraction pattern of chicken tendon collagen. The obtained SAXS patterns were converted into 1D profiles, scattering intensity *I* vs scattering vector *q*, by circular averaging. The scattering vector is defined as:

[3]
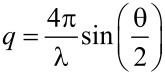


### Mechanical measurements

Obtained silica nanocomposite polyrotaxane gels were cut into cylindrical shapes of 10 mm diameter and 3 mm thickness. All measurements were conducted with a strain-controlled oscillatory rheometer (RSAIII, TA Instruments) using a parallel plate geometry at room temperature. Frequency sweeps were conducted from 0.01 to 80 Hz, applying 1% of the oscillatory compressive strain amplitude, which was still within the range of linear viscoelasticity. Stress relaxation tests were also performed by the compression mode. Stress–compression curves were obtained at a sufficiently slow and constant rate of strain: 0.2%/s.
